# Role of Short Chain Fatty Acids in Controlling T_regs_ and Immunopathology During Mucosal Infection

**DOI:** 10.3389/fmicb.2018.01995

**Published:** 2018-08-24

**Authors:** Natarajan Bhaskaran, Cheriese Quigley, Clarissa Paw, Shivani Butala, Elizabeth Schneider, Pushpa Pandiyan

**Affiliations:** Department of Biological Sciences, School of Dental Medicine, Case Western Reserve University, Cleveland, OH, United States

**Keywords:** Th17, T_reg_, Foxp3, SCFA, *Candida*, oral-microbiome, oral-mucosa

## Abstract

Interactions between mucosal tissues and commensal microbes control appropriate host immune responses and inflammation, but very little is known about these interactions. Here we show that the depletion of resident bacteria using antibiotics (Abx) causes oral and gut immunopathology during oropharyngeal candidiasis (OPC) infection. Antibiotic treatment causes reduction in the frequency of Foxp3+ regulatory cells (T_regs_) and IL-17A producers, with a concomitant increase in oral tissue pathology. While *C. albicans* (CA) is usually controlled in the oral cavity, antibiotic treatment led to CA dependent oral and gut inflammation. A combination of short chain fatty acids (SCFA) controlled the pathology in Abx treated mice, correlating to an increase in the frequency of Foxp3+, IL-17A+, and Foxp3+IL-17A+ double positive (T_reg_17) cells in tongue and oral draining lymph nodes. However, SCFA treatment did not fully reverse the gut inflammation suggesting that resident microbiota have SCFA independent homeostatic mechanisms in gut mucosa. We also found that SCFA potently induce Foxp3 and IL-17A expression in CD4+ T cells_,_ depending on the cytokine milieu *in vitro*. Depletion of T_regs_ alone in FDTR mice recapitulated oral inflammation in CA infected mice, showing that Abx mediated reduction of T_regs_ was involved in infection induced pathology. SCFA did not control inflammation in T_reg_ depleted mice in CA infected FDTR mice, showing that Foxp3^+^ T cell induction was required for the protective effect mediated by SCFA. Taken together, our data reveal that SCFA derived from resident bacteria play a critical role in controlling immunopathology by regulating T cell cytokines during mucosal infections. This study has broader implications on protective effects of resident microbiota in regulating pathological infections.

## Introduction

Dysbiosis of resident microbes is unequivocally associated with immune-related disorders and opportunistic and pathogenic infections (Gallo and Nakatsuji, 2011; Abt et al., 2012; Flint et al., 2012; Hill et al., 2012; Abt and Artis, 2013; Hill and Artis, 2013; Kamada et al., 2013a,b; Spasova and Surh, 2014). Resident microbes remain in direct contact with mucosal surfaces, where both entities are in continuous and complex interactions to achieve immune homeostasis and pathogen clearance (Macpherson and Harris, 2004; Karin et al., 2006; Belkaid and Naik, 2013; Brito et al., 2013; Huffnagle and Noverr, 2013; Blander et al., 2017). Qualitative and quantitative regulation of such interactions is critical for effective immunity without causing excessive inflammatory responses (Bilate et al., 2016; Calderon-Gomez et al., 2016; Hegazy et al., 2017; Navabi et al., 2017). Thus resident microbiota sustain continuous feedback loop mechanisms with mucosal surfaces, shaping mucosal immunity, and mechanisms of inflammation and tolerance in the gut. For example, epithelial glycosylation regulated by resident microbes and group three innate lymphoid cells (ILC3s) control commensal host symbiosis and anti microbial host responses (Goto et al., 2014, 2016). Apart from innate immune responses and Immunoglobulin A (IgA) responses, T cells also play critical roles in these processes. While resident bacteria specific T cells induce colitis, regulatory T cells (T_regs_) control gut immunopathology (Pandiyan et al., 2007, 2011b; Izcue et al., 2009). Commensal microbiota have been shown to direct the development and maintenance of intestinal Th17 and T_reg_ cells (Ivanov et al., 2009; Littman and Pamer, 2011; Atarashi et al., 2013; Furusawa et al., 2013; Smith et al., 2013; Singh et al., 2014; Bilate et al., 2016). With regards to T_regs_, there are a few different populations with variable stability of Foxp3 expression: thymically derived tT_regs_, peripherally derived pT_regs_, and those secreting anti-inflammatory cytokines, such as IL-10 (Tr1) and TGF-β (Th3). Foxp3+ pT_regs_ are not found in the thymic environment but, rather, seem to be induced in peripheral tissues such as gut, in a process that is dependent on resident microbiota. For example, commensal *Bacteroides fragilis* polysaccharide promotes TLR2 dependent development of IL-10 producing T_regs_ and tolerance in experimental colitis model (Round et al., 2011). *Clostridium* sp. promotes the development of Foxp3 T_regs_ in colon by inducing TGF-β production in intestinal epithelial cells (Atarashi et al., 2013). Lastly, resident microbiota derived short chain fatty acids (SCFA) are shown to induce Foxp3 expression in CD4+ T cells, and promote function of Foxp3+T_regs_ and IL-10 producing T_regs_ in intestinal lamina propria (Round et al., 2011; Arpaia et al., 2013; Atarashi et al., 2013; Smith et al., 2013). Anti-inflammatory effects of SCFAs are known to be partly by inhibition of histone deacetylase (HDAC) activity (Vinolo et al., 2011). Supporting this mechanism, even chemical HDAC inhibitors promote T_reg_ functions, and have beneficial effects on allograft survival and autoimmune diseases (Beier et al., 2011; Edwards and Pender, 2011; Arpaia et al., 2013).

In the context of pathogenic infections, resident microbiota have been shown to enhance anti-microbial resistance in some settings, and trigger autoimmune disease development in others, likely depending on qualitative and quantitative responses of T cells (Ivanov et al., 2009; Lee et al., 2011). These studies indicate that resident microbiota is involved in optimal responses, and sometimes exaggerated autoimmune responses, by directly or indirectly modulating T cells through distinct mechanisms. However, a vast majority of these studies has focused on intestinal mucosae and their interactions with gut microbiota, and little is known about host commensal interactions in oral mucosal microenvironment colonized with millions of commensal microbes (Aas et al., 2005; Ghannoum et al., 2010; Ahn et al., 2011), that may be functionally distinct from the gut (Avila et al., 2009; Zaura et al., 2009; Ghannoum et al., 2010; Belda-Ferre et al., 2012; Dang et al., 2012; Yamazaki et al., 2012). Although emerging studies highlight how poly–microbial interactions impact the pathogenesis of oral diseases (Shirtliff et al., 2009; Peters et al., 2012; Wright et al., 2013; Guo et al., 2014; Murray et al., 2014; Xu et al., 2014), the direct effect of such interactions on host immune cells is less clear. Oral infections and inflammation have a dramatic impact on overall human health and have been related adversely to cancer and cardiovascular disease (Karin et al., 2006). Therefore studies focusing on how resident microbes govern oral immune homeostasis are urgently needed. Perturbations in host-commensal homeostasis and Th17 cell/T_reg_ imbalance are associated with oropharyngeal candidiasis (OPC) infections and periodontitis (Milner et al., 2008; Blaschitz and Raffatellu, 2010; Darveau, 2010; Garlet et al., 2010; Kanwar et al., 2010; Li et al., 2011; Pion et al., 2013; Huppler et al., 2012; Cheng et al., 2014). However, the inter-relationship between commensal bacteria and host immune cells in oral disease pathogenesis is unclear. OPC is an opportunistic infection caused mainly by *C. albicans*, a commensal fungus that asymptomatically colonizes ∼60% of healthy individuals. Cancer treatments, genetic causes, and immunodeficiency (as in HIV infection) pre-dispose individuals to chronic mucocutaneous candidiasis (CMC) infections (Marodi and Johnston, 2007; Cheng et al., 2012), causing secondary complications including squamous cell carcinoma and aneurysms (Huppler et al., 2012). While antibiotics are long known to increase the infectivity by *Candida* in humans and mice (Kennedy and Volz, 1985a,b; Nord and Heimdahl, 1986; Nord et al., 1986; Kennedy et al., 1987; Pultz et al., 2005; Peleg et al., 2010), mechanisms are not fully explored. We and others have previously shown a mechanism by which oral T_regs_ play protective roles in anti-candidal host defense and immunomodulation, by promoting IL-17A and controlling TNF-α respectively (Pandiyan et al., 2011a; Carvalho et al., 2012; Whibley and Gaffen, 2014; Whibley et al., 2014; Bhaskaran et al., 2015a,b). We have also shown a mechanism of T_reg_ homeostasis during OPC infection (Bhaskaran et al., 2016). Here we determined whether bacterial SCFA control fungal infection and immunopathology, by increasing the abundance and function of T_regs_ *in vivo*. Our results demonstrate that SCFA produced by commensal microbiota play an important role in combating oral mucosal *Candida* infection. While SCFA mediated an incomplete protective effect in antibiotic treated mice, the partial protective effect required an optimal induction and/or maintenance of T_regs_ and Th7 responses in oral mucosa.

## Materials and Methods

### Mice

C57BL/6 and Foxp3^DTR^ (FDTR) mice were purchased from Jackson Laboratories. All mice were maintained at the CWRU animal facility, cared for and used for experiments in accordance with institutional guidelines under IACUC approved protocols.

### Reagents and Antibodies

Purified α-CD3 (145-2C11), purified α-CD28 (37.51), α-IL-4 (11B11), and α-IFN-γ (XMG1.2) antibodies, ELISA and flow cytometric Foxp3, IL-17A, IFN-γ, and IL-17A antibodies were purchased from Affymetrix/Thermo Fisher Scientific. Recombinant IL-6, IL-1β, IL-12, IL-4, and IL-7 were purchased from (BioBasic Inc., Amherst, NY, United States). Recombinant mouse IL-23 and human TGF-β1 were purchased from R&D systems or Affymetrix/Thermo Fisher Scientific. Mouse cells were cultured in complete RPMI-1640 (Hyclone) supplemented with 10% FCS, 100 U/ml penicillin, 100 μg/ml streptomycin, 2 mM glutamine, 10 mM HEPES, 1 mM sodium pyruvate, and 50 μM β-mercaptoethanol.

### Antibiotics (Abx) and SCFA Treatment *in vivo*

We used age (4 weeks), and gender matched specific pathogen free mice for the experiments. We used both male and female mice for all the experiments. For the depletion of SCFA producing anaerobic resident bacteria we used the antibiotic regimen and time-line that were reported previously (Arpaia et al., 2013; Furusawa et al., 2013; Smith et al., 2013). We administered a cocktail of ampicillin; 500 mg/L, vancomycin; 500 mg/L and metronidazole; 1 g/L in the drinking water and monitored the fluid intake regularly. We continued the pretreatment with Abx for about 4 weeks before the mice were orally infected with CA. To examine the effect of SCFA, some mice received a mixture of sodium propionate and sodium butyrate (Millipore-Sigma; 50 mM each) in drinking water. This concentration has been previously shown to deplete anaerobic bacteria in the gut (Arpaia et al., 2013; Furusawa et al., 2013; Smith et al., 2013). According to these studies, the amounts of SCFA provided in the drinking water were in the range of 35–150 mM, where they observed significant effects on T_regs_. Moreover, SCFAs are found at concentrations ranging from 50 to 100 mM in the colonic lumen (Cummings et al., 1987). In the oral cavity, human salivary concentrations of concentrations of SCFA are 0.5–5 mM (Das et al., 2015). Thus we rationalized to use these concentrations in drinking water in our studies. As SCFAs were given as sodium salts, pH matched sodium water was given to control mice. As additional controls, some mice were administered with Abx but were not infected. Some were given immunosuppressive cortisone in some experiments (data not shown).

### OPC Infection in Mice

Age and gender matched C57BL/6 and FDTR transgenic mice were infected as previously described (Kamai et al., 2001; Conti et al., 2009; Pandiyan et al., 2011a). Briefly, they were infected under anesthesia with a mixture of ketamine/xylazine, by placing a cotton ball saturated with 2 × 10^7^ *C. albicans* (CA) (SC-5314) blastospores sublingually for 90 min. In some experiments, they were immunosuppressed with 225 mg/kg cortisone acetate. To model recurrent infection, we infected with CA, and on day 7 after initial infection we performed re-infection. PBS inoculated mice were used as uninfected sham control as described previously (Conti et al., 2009). For experiments involving FDTR transgenic mice that express diphtheria toxin (DT) receptor in T_regs_, we intraperitoneally injected DT (20 μg/10 gm body weight) on days -6, -3, -1, 0, 3, and 6 of infection. We monitored the weight regularly after infection until euthanasia.

### Cell Purification

Splenocytes and lymphnodes were harvested from 5 to 8 weeks old mice. CD4^+^ naïve cells were purified using Easysep naïve CD4 isolation kits purchased from Stemcell technologies (Vancouver, BC, Canada).

### Th Differentiation *in vitro*

For Th17 polarizing condition, we stimulated CD4^+^ CD44^low^ CD62L^high^ CD25^-^ naïve cells using 1 μg/ml soluble α-CD3 and 2 μg/ml soluble α-CD28, 25 ng/ml IL-6, 2 ng/ml TGF-β, 6 μg/ml α-IFN-γ, and 6 μg/ml α-IL-4 antibodies in the presence of T cell depleted antigen presenting splenocyte cells (APC). For iT_reg_ polarizing condition we stimulated the naïve cells using 1 μg/ml soluble α-CD3 and 2 μg/ml soluble α-CD28 with 20 ng/ml IL-2, 4 ng/ml TGF-β in the presence of APC. Naïve cells were magnetically sorted from spleen (SPLN) and lymphnodes (LN) using Stemcell Technologies naïve cell sorting kits. When indicated, cells from whole SPLN and LN, or indicated mucosal tissues were used for stimulation under Th17 or iT_reg_ polarizing conditions as above. When indicated, we added fresh stocks of SCFA (Sigma-Aldrich) namely, sodium acetate (10 mM), sodium propionate (P) (1 mM) and sodium butyrate (B) (0.1 mM) at the same time as stimulation. As acetate did not show consistent effects in our preliminary experiments, we focused only on propionate and butyrate in subsequent experiments. We determined the above concentrations based on titrations in T cell cultures and concentrations used in previous literature. We kept the concentration ranges close to physiological SCFA concentrations in human saliva (Yu et al., 2014). We found that propionate and butyrate, when used at concentrations higher than above, completely blocked the proliferation and activation of T cells. pH matched sodium salt were used as untreated control.

### Histology and Intracellular Staining of Cytokines

For immunocytochemical Periodic Acid Schiff (PAS) staining, tissues were washed with PBS, fixed with 10% formalin overnight and suspended in 70% ethanol to prevent hyperfixation. Paraffin sectioning and staining of paraffin sections were performed by Histoserv, Inc., Germantown, MD, United States. For single-cell staining, cells were isolated from mice, washed in PBS, fixed with Fix-Perm kit (eBiosciences/Thermo Fisher Scientific, Waltham, MA, United States). Before fixation, the cells were stimulated with PMA (50 ng/ml) and ionomycin (500 ng/ml) for 4 h, with brefeldin-A (10 μg/ml) added in last 2 h.

### Flow Cytometry

All data were acquired using BD FACS Fortessa cytometer and were analyzed using FlowJo 9.6 software.

### Statistical Analyses

*P*-values were calculated by non-parametric Mann–Whitney test in Prism 6.0 (GraphPad Software, Inc.).

## Results

### Abx Mediated Depletion of Resident Bacteria Reduces Resistance to Oral *Candida albicans* Infection, and SCFA Administration Partially Restores Protective Host Defense

**FIGURE 1:**
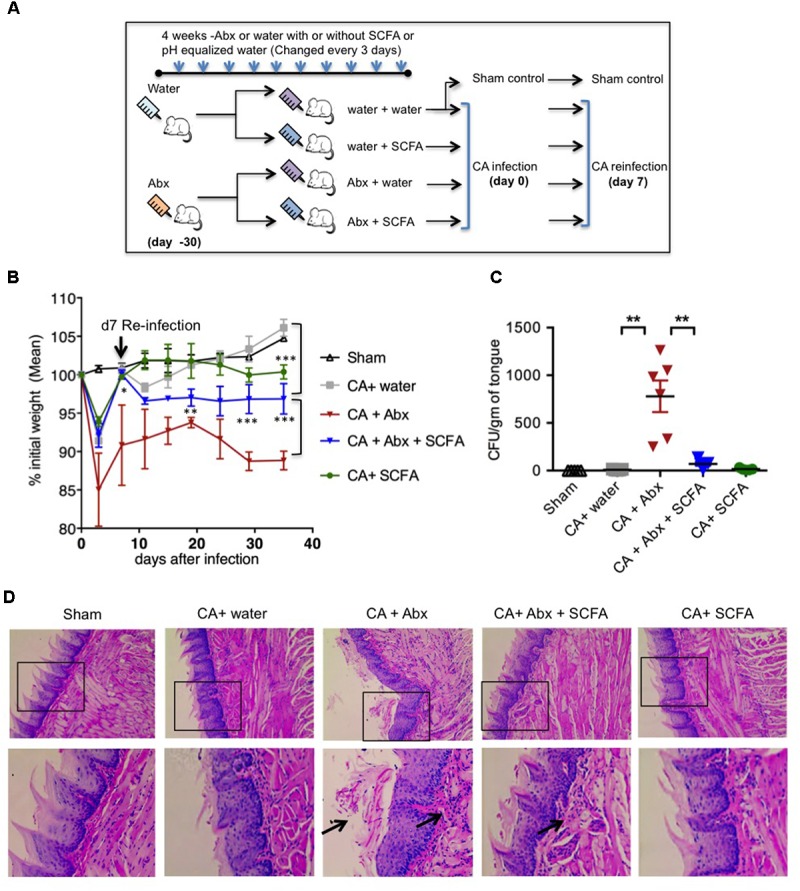
Abx treatment results in impaired resistance to OPC in mice, while SCFAs reduce fungal burden and immunopathology. **(A)** Abx and SCFA administration schedule. Abx, antibiotics; SCFA, short chain fatty acids. **(B)** Abx or SCFA treated C57BL/6 mice were orally infected and re-infected with *C. albicans* (CA) *n* = 4/group. Body weight was measured every 3rd day until the day of sacrifice. **(C)** On day 28 or 29 after secondary infection, we re-infected the mice. Three days after 2nd re-infection, we isolated the tongue, and determined the fungal growth, by plating the tongue lysates in Sabaroud dextrose agar. Fungal Colony Forming Units (CFU)/gm of tongue tissue plated in 10-fold serial dilutions and assessed in triplicates. Mean values ±SEM are plotted. **(D)** Three days after 2nd re-infection, we fixed the intact tongues and performed PAS staining and microscopic examination (200× magnification). Bottom panels show magnified images of respective upper panels. Data are representative of at least four experiments. ^∗^*P* < 0.05, ^∗∗^*P* < 0.005, and ^∗∗∗^*P* < 0.0005.

Abx have been shown to reduce SCFA producing commensal anaerobic bacteria previously (Arpaia et al., 2013; Furusawa et al., 2013; Smith et al., 2013). Therefore, we hypothesized that Abx mediated reduction of SCFA levels will increase fungal infectivity and immunopathology during OPC. To that end, we depleted SCFA producing anaerobic resident bacteria using the similar method and time-line reported previously (Arpaia et al., 2013; Furusawa et al., 2013; Smith et al., 2013). We administered a cocktail of antibiotics (Abx), namely ampicillin; 500 mg/L, vancomycin; 500 mg/L and metronidazole; 1 g/L) in drinking water from weaning (∼4 weeks of age) and monitored the fluid intake regularly. We continued the pretreatment with Abx for about 4 weeks before the mice were orally infected (**Figure 1A**). We found that the combination of Abx we used significantly diminished salivary SCFA levels in oral mucosa (**Supplementary Figure S1A**). To examine the effect of SCFA, some mice received a mixture of sodium propionate and sodium butyrate (50 mM each) in drinking water (Arpaia et al., 2013; Furusawa et al., 2013; Smith et al., 2013). As SCFAs were given as sodium salts, pH matched sodium water was given to control mice. To model recurrent infection, we infected with CA, and on day 7 after initial infection we performed re-infection. PBS inoculated mice were used as uninfected sham control as described previously (Conti et al., 2009). As additional controls, some mice were administered with Abx but were not infected. To determine SCFA effects on *Candida* (CA) growth, we plated CA *in vitro* with different SCFA, and administered SCFA as above in OPC mice, and found that SCFA did not control CA growth directly *in vitro* and *in vivo* (**Supplementary Figure S1B**). As positive controls for infection, some mice were given immunosuppressive cortisone, which succumbed to severe fungal infection and weight loss and had to be euthanized after primary infection (**Supplementary Figures S1B**, **S2**). We monitored the weight regularly until day 28 or 29 after infection, at which point the mice were euthanized. We observed that administration of Abx rendered mice significantly more susceptible to weight loss during primary infection and reinfection (**Figure 1B**). Mice treated with Abx + SCFA completely regained their weight during primary infection. Also during reinfection, this group of mice partially but significantly recovered from weight loss compared to mice that were treated with Abx alone. As expected, uninfected mice with and without Abx did not lose weight, but rather gained weight (data not shown, **Figure 1B**). On day 28 or 29 after secondary infection, we re-infected the mice and isolated the tongue, and determined the fungal burden using tongue lysates, as well as PAS staining and histo-pathological examination of intact tongue tissues 3 days later. We found that the mice receiving Abx showed higher fungal burden in lysates, and revealed fungal hyphae (**Figures 1C,D**). Mice that received Abx and SCFA showed significantly lower levels of fungal hyphae, comparable to infected mice that received only water. Control uninfected mice, as well as infected mice that received no Abx, showed no fungus in their tongues (**Figures 1C,D**). These data show that Abx caused excessive tissue pathology, and SCFA promotes fungal clearance and inflammation resolution during recurrent OPC infection in Abx treated mice.

### Abx Mediated Depletion of Resident Bacteria Reduces IL-17A Production and T_reg_ Frequencies During OPC Infection, and Administration of SCFA Partially Restores Th17 and T_reg_ Cells

**FIGURE 2:**
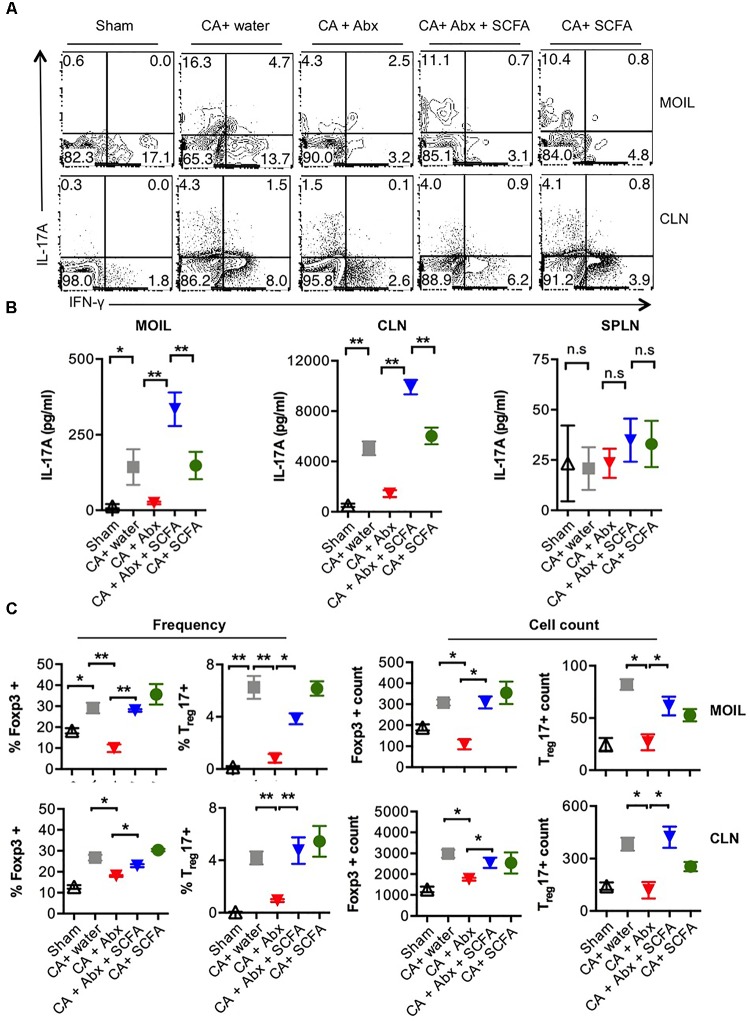
Abx treatment leads to Th17 and T_reg_ cell reduction, and SCFAs restore these cell populations during OPC. **(A)** Mice were treated with Abx and SCFA, and infected as in **Figure 1** (*n* = 4/group). On day 1 after second infection, cells isolated from tongue (MOIL) and draining CLN and were re-stimulated before cytokine analyses. Each FACS plot represents individual mouse in a group. Flow cytometric contour plots showing expression of IFN-γ and IL-17A (all gated on CD4+ cells). **(B)** ELISA quantification of IL-17A in supernatants collected from MOIL and CLN cultures re-stimulated as in **(A)**. **(C)** MOIL and CLN cultures re-stimulated as in A for cytokine assessment. Statistic analyses on Foxp3+ and Foxp3+IL-17A+ cells. Mean values from four individual data points (mice)/group are shown. Note the differences in “Y-axis.” Statistical analyses was done using Mann–Whitney test (^∗^*P* < 0.05 and ^∗∗^*P* < 0.005). Data represent triplicate experiments.

Because of the known host-protective functions of T_reg_ and Th17 cells (Pandiyan et al., 2011a; Carvalho et al., 2012; Whibley and Gaffen, 2014; Whibley et al., 2014; Bhaskaran et al., 2015a,b), we then determined the expression of IL-17A and Foxp3 in CD4 T cells recovered from these mice. On day 28 or 29 after infection, we re-infected the mice, and a day later isolated the mouse oral intra-epithelial and lamina propria leukocytes (MOIL) of tongue and draining cervical lymph nodes (CLN), evaluated the frequency of Th17 cells and T_regs_ using flow cytometry and ELISA, after a short *in vitro* restimulation. Quantification of IL-17 cytokine levels in CD4 cells from infected mice revealed that Abx significantly decreased frequency of IL-17A producers among CD4 cells compared to mice that did not receive Abx (**Figure 2A**, 2nd and 3rd panels). Also, Abx caused a significant reduction in IFN-γ producers (**Figure 2A**). SCFA increased the frequency of IL-17A producers, but not the IFN-γ producers, implying that SCFA specifically promotes IL-17A production in T cells in the context of OPC (**Figure 2A**, 4th panel, **Supplementary Figure S3**). ELISA quantification corroborated these intracellular cytokine staining data and also confirmed that CA mediated IL-17A responses and Abx mediated effects on IL-17A were restricted to oral mucosa and draining lymph nodes and not observed in spleen (**Figure 2B**). Evaluation of CD4+Foxp3+ cell frequencies revealed that CA infection induces an increase in Foxp3+ cell frequencies and cell numbers in MOIL and CLN, and that Abx prevented the T_reg_ induction (**Supplementary Figure S4**, 2nd and 3rd panels, **Figure 2C**). Furthermore, we observed that Abx also reduced the frequency of preexisting Foxp3+ cells in MOIL. Importantly, SCFA administration significantly and completely restored the Foxp3+ cell frequencies to levels of mice without Abx treatment (**Supplementary Figure S4**, 4th panel, **Figure 2C**). These data show that while Abx depletes Th17 and T_reg_ cells in oral mucosa, SCFA increases IL-17A production as well as T_reg_ frequencies in CD4+ T cells during OPC infection in Abx treated mice.

### Abx Treatment Also Results in Gut Inflammation During OPC Infection in Mice

**FIGURE 3:**
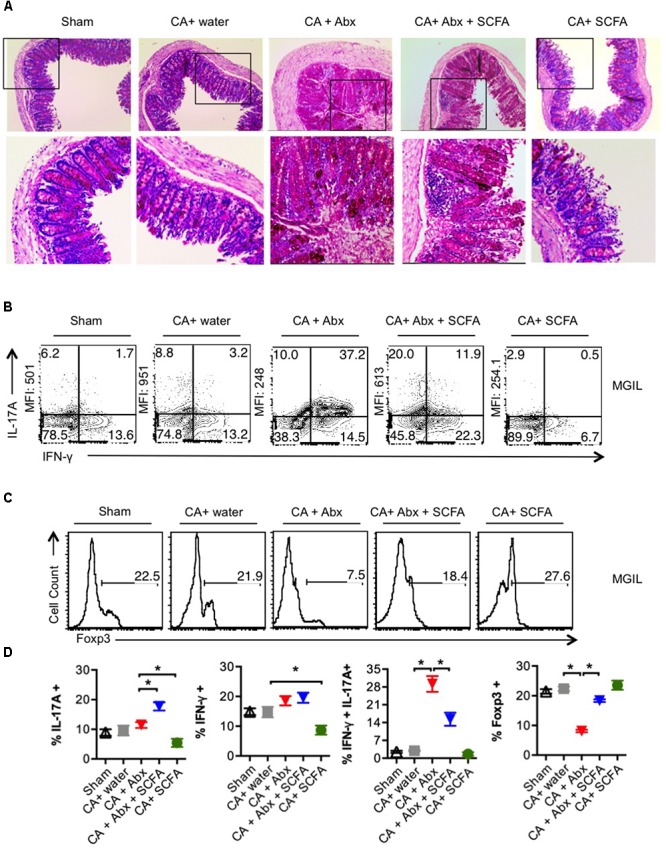
Abx treatment induces colon inflammation during OPC in mice, while SCFAs partially reduce immunopathology. **(A)** Three days after 2nd re-infection, we isolated and fixed colon tissues and performed PAS staining and histo-pathological examination using microscopy (200× magnification). **(B,C)** One day after second re-infection, MGIL were isolated from colon and re-stimulated as in **Figure 2**. Flow cytometric contour plots show the expression of IFN-γ and IL-17A **(B**), and Foxp3 **(C)**. Mean fluorescent intensities (MFI) of IL-17A expression are shown on the “Y- axes.” **(D)** Statistical analyses for % cytokine + cells or % Foxp3+ in MGIL (*n* = 4/group) was performed using Mann–Whitney test (^∗^*P* < 0.05). Mean ± SEM are shown. At least three independent experiments showed similar results.

SCFA facilitated restoration of Th17 cells and T_reg_ cells and infection clearance, but did not reverse weight loss. These results prompted us to examine the gut, as gut inflammation is known to cause weight loss. On day 28 or 29 after secondary infection, we re-infected the mice and assessed gut inflammation as well as the presence of fungal hyphae using histopathological PAS staining 3 days later. We observed that the colon of the infected mice treated with Abx had considerable thickening of colon wall, epithelial cell hyperplasia, elongated crypts compared to infected mice that received no Abx (**Figure 3A**). While we did not observe the presence of fungal hyphae, PAS staining of colon sections revealed increased glycoproteins including mucin in mice that received Abx. One day after second re-infection, we isolated colon to obtain single cell suspensions mouse gut intraepithelial and lamina propria leukocytes (MGIL). Flow cytometry analyses of MGIL revealed that Abx recipients contained a clearly increased population of inflammatory IL-17A- and IFN-γ producing T cells (Th1^∗^) compared to the other groups (**Figure 3B**, 3rd panel, **Figure 3D**). However the mean fluorescent intensity (MFI) of IL-17A expression in IL-17A+ cells was somewhat much lower than the other groups (**Figure 3B**, 3rd panel, **Figure 3D**). SCFA administration considerably increased the MFI of IL-17A cytokine expression and the frequency of Th17 cells (that produced IL-17A only). While SCFA did not affect IFN-γ+ producers (Th1 cells) in Abx treated mice, it significantly decreased the frequency of IL-17A+IFN-γ+ double producers (Th1^∗^ cells) in the gut. In contrast to the oral mucosa, and correlating to gut inflammation, the overall frequency of IFN-γ producers significantly increased with Abx, while SCFA administration reduced IFN-γ production in CD4+ T cells in colon (**Figure 3B**, 3rd and 4th panels, **Figure 3D**). Consistent with the results in oral mucosa, we found that Abx dramatically reduced the size of T_reg_ population in gut, and was partially but significantly restored by SCFA (**Figures 3C,D**). These data show that Abx mediated depletion of gut resident microflora leads to reduction of Foxp3+ T_reg_ and Th17 cells, but a concomitant increase in IFN-γ production in colonic CD4+ T cells in colon as well gut immunopathology. These effects were only partially reversed by SCFA. Altogether, SCFA confers only partial protection to gut inflammation caused by oral CA infection in the context of resident microbiota depletion.

### SCFA Promotes Th17 Induction and T_reg_ Cell Induction and/or Expansion *in vitro*

**FIGURE 4:**
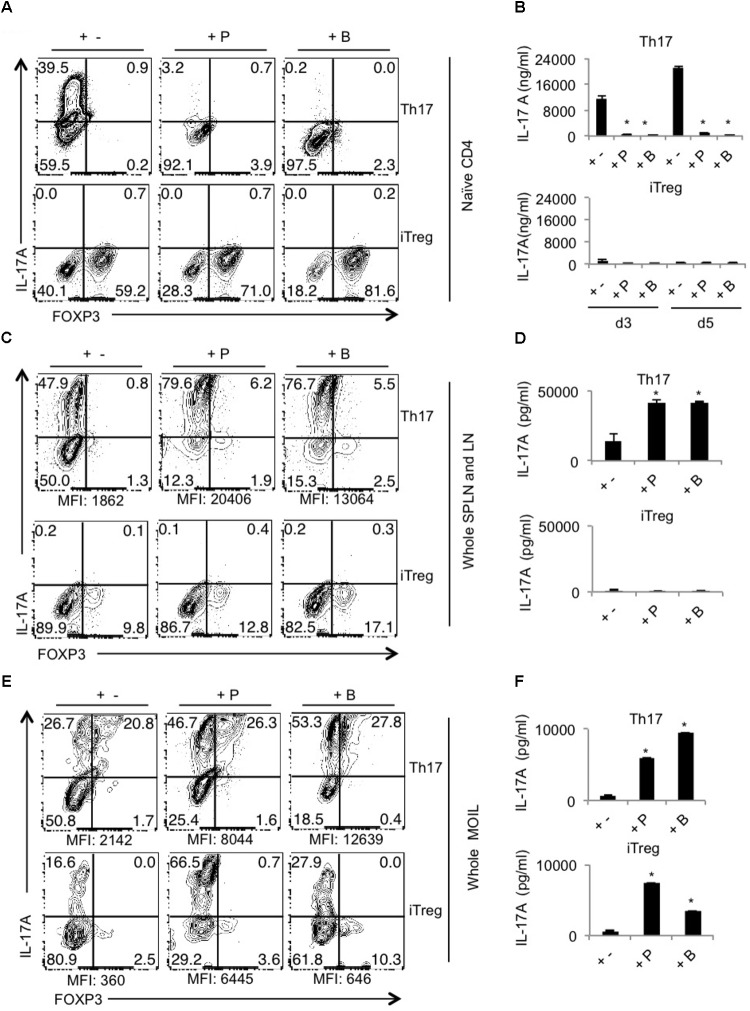
SCFAs promote IL-17A and Foxp3 expression, depending on cytokine milieu *in vitro.* Naïve cells from normal WT mice were isolated from pooled SPLN and all lymph nodes. They were stimulated with T cell depleted APC under Th17 conditions (upper panel) or iT_reg_ polarization conditions (lower panel) with or without propionate (P) and butyrate (B). **(A)** IL-17A and Foxp3 expression (in CD4+ cells) were assessed by flow cytometry on day 4 after stimulation. **(B)** Supernatants were collected on days 3 and 5, and IL-17A levels were measured by ELISA. **(C,D)** Single cells suspensions were derived from pooled SPLN and all lymph nodes and were stimulated under Th17 conditions (upper panel) or iT_reg_ polarization conditions (lower panel) with or without propionate (P) and butyrate (B). Flow cytometric assessment of IL-17A and Foxp3 expression on day 4 are shown in **(C)**. ELISA quantification of IL-17A in culture supernatants on day 4 is shown in **(D)**. (**E,F**) Single cells suspensions were derived from MOIL and were stimulated under Th17 conditions (upper panel) or iT_reg_ polarization conditions (lower panel) with or without propionate (P) and butyrate (B). Flow cytometric assessment of IL-17A and Foxp3 expression on day 4 are shown in **(E)**. ELISA quantification of IL-17A in culture supernatants on day 4 is shown in **(F)**. ^∗^*P* < 0.05, as measured by Mann–Whitney tests. Data represent three independent experiments. Geometric mean fluorescence intensities of IL-17A expression in CD4+ cells are shown as MFI in flow cytometric plots in **(C,E)**.

Next we determined whether SCFA promotes Th17 and T_reg_ differentiation *in vitro* in a manner similar to what we observed *in vivo*. We first sought to examine the effects on naïve CD4+ T cells stimulated under Th17 (**Figure 4A**, upper panel) and iT_reg_ skewing conditions (**Figure 4A**, lower panel; Korn et al., 2009). We assessed the IL-17A production on day 4 after stimulation of naïve CD4 T cells under Th17 polarizing conditions. We were surprised to find that both propionate and butyrate did not promote but rather inhibited Th17 differentiation of naïve cells (**Figure 4A**, upper panel). To test whether it was time-kinetics differences, we measured IL-17A at different time-points. Supernatants collected and day 3 and 5, only confirmed the repressive effects of SCFA in Th17 differentiation (**Figure 4B**, upper panel). However, similar to *in vivo* data, both SCFA increased the frequency of Foxp3+ cells (*in vitro* induced T_regs_) when naïve cells stimulated under iT_reg_ conditions, without inducing IL-17A (**Figures 4A,B**, lower panels). For the differentiation of naïve cells in to Th17 cells, we used T cell depleted spleen as APC. Because SCFA did not enhance, but rather inhibited Th17 cell differentiation in CD4^+^ naïve cells, we hypothesized that SCFA may increase Th17 cells *in vivo*, only in the presence of certain other APC, or other activated T cells. Therefore we tested the effects of SCFA on CD4 T cells in single cell suspension derived from whole spleen and lymphnodes *in vitro.* We activated the cells from whole lymphoid organs under Th17 and iT_reg_ cytokine milieu as above and determined Foxp3 and IL-17A expression. We found that SCFA significantly boosted the frequency of Th17 cells and IL-17A levels in supernatants, as determined by flow cytometry and ELISA, respectively (**Figures 4C,D**, upper panels). There were a very few Foxp3^+^ cells in these Th17-polarized cultures, but were unchanged with SCFA treatment. However, the frequency of IL-17A^high^ expressing Foxp3+ (T_reg_17) cells increased with SCFA treatment (**Figure 4C**, upper panel, X-axis). When cells stimulated under iT_reg_ conditions were examined, we found that SCFA, especially butyrate induces Foxp3 expression, although only moderately (**Figure 4C**, lower panel, X-axis). As expected, SCFA did not up-regulate IL-17A in iT_reg_ SPLN and LN cultures (**Figure 4D**, lower panel). To validate the effects of SCFA on T cells in oral mucosal tissues that was observed during OPC infection *in vivo*, we next stimulated oral mucosal T cells in the presence of SCFA *in vitro*. We stimulated MOIL under polarizing conditions for 4 days and examined Foxp3 and IL-17A expression as above. As in whole lymphoid organ cell suspension cultures, we found that SCFA significantly heightened both the frequency and geometric MFI of IL-17A expression in CD4+ cells under Th17 polarization conditions (**Figures 4E,F**, upper panels, **Supplementary Figure S5**). Importantly, MOIL harbored IL-17A^high^ expressing Foxp3+ cell population (T_reg_17), whose frequency were further increased in SCFA treated cultures (**Figure 4E**, upper panels, **Supplementary Figure S5**). Surprisingly, under iT_reg_ polarization conditions, propionate and butyrate enhanced the frequencies of both FoxP3 and IL-17A expressing CD4+ T cells, without giving rise to T_reg_17 cells. The effects of propionate were only moderate in increasing Foxp3+ cells in iT_reg_ cultures (**Figure 4E**, lower panels, **Supplementary Figure S5**). Supernatants derived from SCFA treated MOIL showed heightened levels of IL-17A both in Th17 and iT_reg_ cultures. Acetate did not affect naïve cell differentiation, but had modest effects in increasing Foxp3+ cells and IL-17A+ cells in whole SPLN/LN and MOIL cultures. Although the effects were reproducible, they were moderate, when compared to propionate and butyrate (data not shown). Taken together, these data demonstrate that while SCFA inhibited Th17 cell differentiation of naïve cells in the presence of T cell depleted splenocytes, they promote the induction and or expansion of IL-17A and Foxp3 expressing CD4 T cells in lymphoid organs and mucosal tissues. These data show that while SCFA can have differential effects on IL-17A expression depending on cellular and cytokine milieu, they increase the frequency of Foxp3+ cells in all culture conditions tested. While their ability to promote the induction of Foxp3+ expression in naïve cells cannot be ruled out in our experiments, it is more likely they expand the pre-existing T_regs_ in CD4+ T cell population.

### SCFA Did Not Protect T_reg_ Depleted FDTR Mice From Tongue Immunopathology During OPC

**FIGURE 5:**
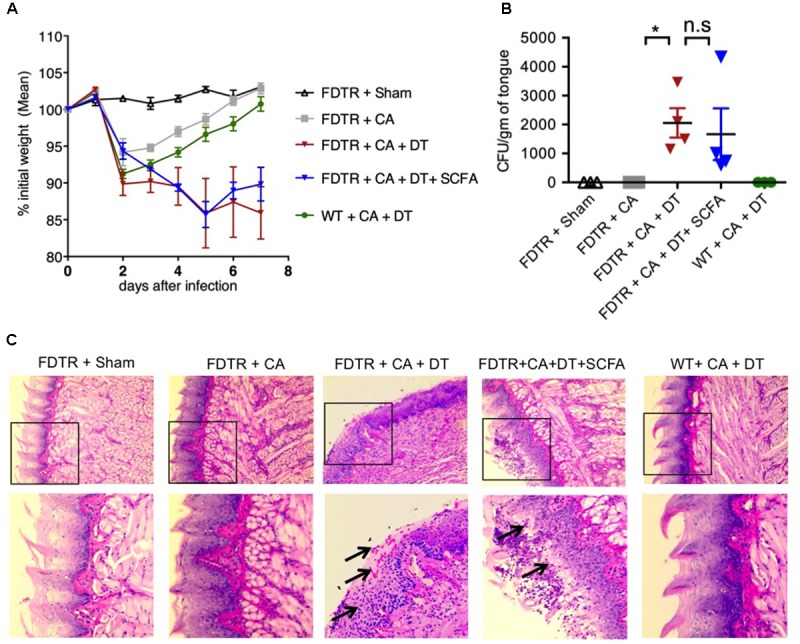
T_regs_ protect CA infected mice by reducing fungal burden and immunopathology. SCFA have little or no impact in fungal burden in T_reg_ depleted FDTR mice. FDTR and WT mice were pre-injected with DT on days -6, -3, -1, 0, 3, and 6 of infection (*n* = 4/group). Some mice received SCFA as in the above regimen (*n* = 4/group). **(A)** The percent weight change in mice with respect to d0 of infection. **(B,C)** On day 7 after infection, mice were re-infected with CA. Tongues were harvested on day 3 after re-infection. CFU/gm of tongue tissue **(B)** and tongue PAS staining (shown in 200× magnification) **(C)**, assessing the fungal burden. Bottom panel in **(C)** shows magnified images of the region marked in the respective upper panels. Mean values ± SEM are plotted. *P*-values (^∗^*P* < 0.05; n.s, non-significant) were measured using Mann–Whitney tests. These data represent three independent experiments showing similar results.

**FIGURE 6:**
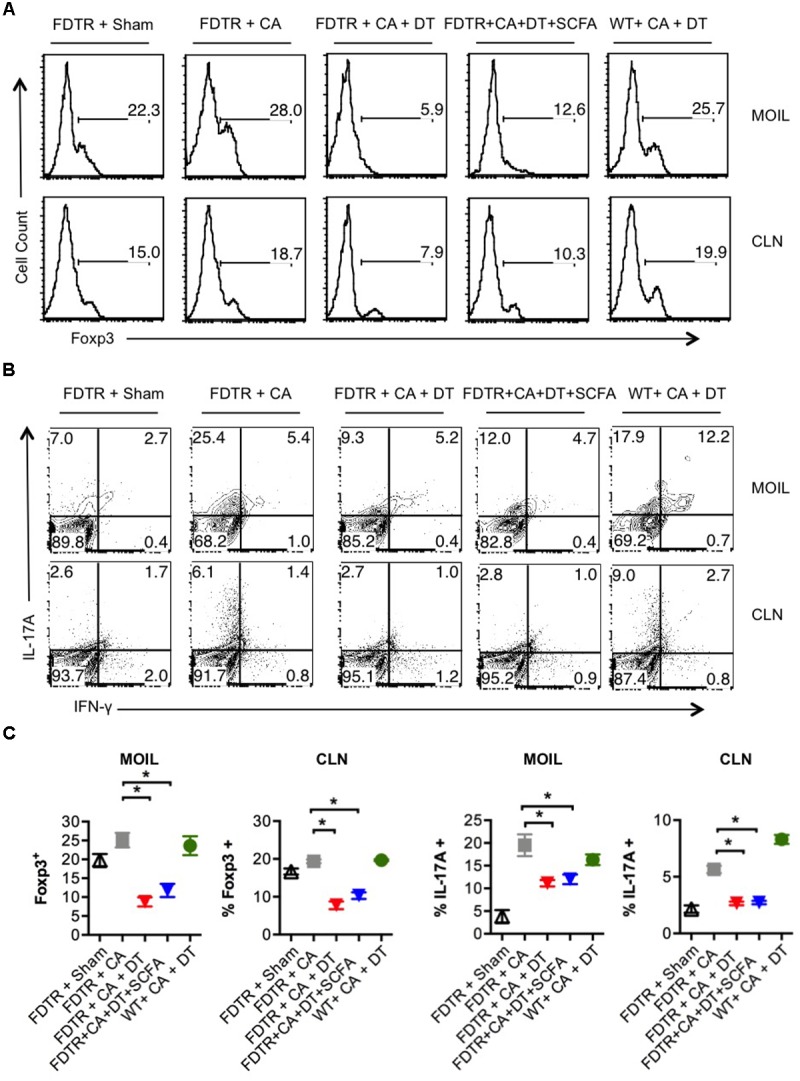
SCFA mediated induction of T_regs_ enhances the frequency of IL-17A producers in CA infected mice. **(A)** FDTR and WT mice were pre-injected with DT, or pretreated with SCFA as in **Figure 5**. On day 7 after infection, mice were re-infected with CA, MOILs and CLN were harvested. Flow cytometric analyses of intracellular staining of Foxp3 expression **(A)**, and IFN-γ and IL-17A in CD4+ T cells are shown **(B)**. **(C)** Statistical analyses for % Foxp3+ or % IL-17A+ cells (*n* = 4/group) was performed using Mann–Whitney test (^∗^*P* < 0.05). Mean ± SEM are shown. Results represent data from 3 independent experiments.

Because our previous studies showed the requirement of T_regs_ for optimal Th17 responses and immunomodulation during OPC infection (Pandiyan et al., 2011a), we hypothesized that Abx mediated depletion of T_regs_ could be a critical factor causing the adverse effect during OPC infection. Also, the above results showing SCFA mediated induction/expansion of T_regs_ both *in vivo* infection and *in vitro* potentially implies that SCFA mediated protective effects is T_reg_ dependent *in vivo*. We decided to explore this possibility more carefully. We devised a more direct experiment to examine whether SCFA affords protection from immunopathology during OPC infection in settings where T_regs_ are absent during acute primary infection. To this end, we employed FDTR transgenic mice in which the DT receptor is expressed in T_regs_, where these cells can be depleted by injecting DT during the course of infection. Because the long-term depletion of T_regs_ by DT is lethal and causes autoimmunity in mice, we did not deplete T_regs_ in the same regimen as above, but only the week before primary infection. One of the DT groups received prior SCFA treatment under the above-mentioned regimen (starting from day -30 of infection, **Figure 1A**), while the other group did not. As controls, we had FDTR sham group, FDTR infected group without DT, and WT group receiving DT. As expected, FDTR infected group that received DT continued to lose weight while infected mice that received no DT, recovered from weight loss starting from day 3 after infection. Interestingly, SCFA did not protect T_reg_ depleted FDTR infected group from weight loss (**Figure 5A**). On day 7 after infection we re-infected the mice with CA and measured immunopathology and infection susceptibility as assessed by tongue PAS staining 3 days after re-infection. Both T_reg_ depleted groups, without and with SCFA treatments showed persistence of fungus in tongue (**Figures 5B,C**). However, unexpectedly, SCFA treated mice had considerably less infiltration of cells, moderately less tongue immunopathology than mice that did not receive SCFA (**Figure 5C**, compare 2nd, 3rd, and 4th panels). DT treated WT mice did not show fungal burden, demonstrating that susceptibility to infection was not caused by DT injection, but rather by possible depletion of T_regs_ in FDTR infected group. To confirm T_reg_ depletion in these mice, we measured the frequency of Foxp3+cells among CD4+ T cells in MOIL and CLN, on day 1 after re-infection. As expected, infected mice showed moderately increased Foxp3+ T_regs_ compared to sham controls, showing that CA infection induces T_regs_ in the periphery (Bhaskaran et al., 2015a; **Figure 6A**, 1st two panels, **Figure 6C**). DT treated groups showed significant depletion of T_regs_, but their frequencies were only slightly increased (but not significantly) in mice under SCFA treatment (**Figure 6A**, 3rd panel, **Figure 6C**). WT mice that received DT injection showed normal levels of T_regs_. When we examined the gut we did not find gross inflammation in any of the groups (**Supplementary Figure S6**). This is likely due to these mice having an intact microbiome combined with short time span of T_reg_ depletion, compared to those with long term infection and Abx treatment in previous experiments (**Figures 1**, **2**). Evaluation of IL-17A and IFN-γ producing CD4+ T cells in MOIL and CLN revealed that T_reg_ depletion caused a considerable decrease in IL-17A production from 25.4 to 9.3% consistent to our previous data (Pandiyan et al., 2011a), showing that T_regs_ are required for optimal IL-17A production during mucosal infection (**Figure 6B**, 2nd and 3rd panels, **Figure 6C**). T_reg_ depletion affected neither the frequency of IFN-γ producers nor the IFN-γ+IL-17A+ double producers (Th1^∗^) in DT injected groups. Importantly, SCFA treatment failed to restore the induction of IL-17A expressing cells during OPC (**Figure 6B**, 4th panel, **Figure 6C**). Consistent with these data, we also found that SCFA administration did not protect Abx treated mice from weight loss in the absence of T_regs_ during short-term acute CA infection (**Supplementary Figure S7**). Taken together, these results reveal that SCFA mediate their protective effects by one or more ways(s) of maintenance, or induction of T_regs_ and Th17 cells during OPC.

## Discussion

Here we studied how SCFA produced by commensal bacteria control T_regs_ in the context of OPC pathogenesis. We found that Abx not only reduced T_reg_ cells, but also the frequency of IL-17A producers among CD4 T cells both in oral mucosal tissues and draining CLN. Reduction of T_regs_ with concomitant reduction of Th17 cells and an increase in tissue pathology and fungal burden is consistent with our previous studies that demonstrated oral T_regs_ play protective roles in anti-candidal host defense and immunomodulation (Pandiyan et al., 2011a; Whibley and Gaffen, 2014; Bhaskaran et al., 2015a,b, 2016). New data presented here demonstrate a surprising protective effect of SCFA on the resolution of OPC infection. It appears that resident microbiota derived SCFA provide molecular signals to induce and, or maintain T_reg_ and Th17 cells that have known protective roles during OPC infection. Interestingly, while Abx treatment reduced both IL-17A and IFN-γ in the oral mucosa, SCFA restored only the expression of IL-17A and Foxp3, and not IFN-γ, showing specific effects of SCFA in Th17 cells and T_reg_ cells in the context of OPC infection and Abx treatment (**Figure 1** and **Supplementary Figure S3**). In fact in infected mice that did not receive Abx, we found that SCFA rather inhibited IFN-γ production without having an adverse outcome (**Figure 2A**, 5th panel, **Supplementary Figure S3**), showing that normal microbiome might have additional protective effects that are dependent on IFN-γ expression and independent of SCFA and T_regs_ in oral mucosa. Mechanistic details underlying the differential effects of SCFA on IFN-γ in oral mucosa and gut mucosa remain to be studied. Although SCFA reversed the pathology caused by Abx, one caveat to our experiments is the lack of direct evidence that Abx resulted in SCFA depletion in mice. We also did not clarify whether there were changes in microbiota in the gut or oral mucosa. However, we have cautiously made our inference that Abx must have caused reduction of resident bacteria and SCFA, based on previous reports that showed SCFA reduction with Abx regimen (Atarashi et al., 2011; Arpaia et al., 2013; Smith et al., 2013). Also, whether SCFA are generated by mouse oral microbiome and if those bacteria were also reduced locally remains to be seen. However, previous studies in humans that showed presence of SCFA in saliva and dys-regulated levels of SCFA in saliva samples of periodontitis patients support the contribution of SCFA by oral resident microbiota (Yu et al., 2014).

As we previously reported, we also found that CA infection by itself increases T_reg_ frequencies (**Figure 2C** and **Supplementary Figure S4**), likely in a TLR-2 dependent manner (Bhaskaran et al., 2015a). We believe that these are peripheral T_regs_ that are induced in local mucosa during infection and likely play immunomodulatory roles to prevent exacerbated inflammation. However, the inter-relationship between tT_regs_ and pT_regs_ in mucosal tissues during an ongoing infection is not clear and remain to be studied. Data presented here do not allow us to distinguish between the possible proliferation (and/or consequent expansion) of IL-17A+, Foxp3+, and T_reg_17 cells and the induction of cell differentiation and consequent expression of these molecules in T cells. Based on absence of naïve cell differentiation in to IL-17A+ and T_reg_17 cells in spleen (**Figure 4A**), we can make a cautious inference that SCFA expand pre-existing IL-17A+ cells in mucosa. The role of microbiome, and SCFA in particular, on epithelial cells and APC in maintaining or inducing these T_regs_, as well as in IL-17A induction in cells other than Th17 cells during infection will be investigated in the future. One of the surprising findings of our study is that Abx in the context of OPC caused excessive inflammation in the gut that showed features of ulcerative colitis. Although PAS staining did not show any presence of visible fungal hyphae in the gut, it revealed excessive mucus production (**Figure 3A**; McManus, 1946; Erben et al., 2014). It is possible that a combination of Abx and OPC infection resulted in overall gut microbial dysbiosis and excessive mucus production. These data are consistent with a previous study that showed an association between inflammatory bowel disease and resident fungal microbiota dysbiosis in humans (Sokol et al., 2017). In our current study, the inflammation in the gut was associated with a significant loss of colon T_regs_ and considerable increase in cells that co-produced IL-17A and IFN-γ (**Figure 3B**, 3rd panel). These cells, known as Th1^∗^ cells, are shown to be associated with inflammatory bowel diseases and autoimmunity in humans (Zielinski et al., 2012; Sallusto, 2016). Th1^∗^ cells that we found in MGIL were IL-17A^low^ producers (with low MFI of IL-17 expression), in contrast to IL-17A^high^ cells that only produced IL-17A (Th17 cells) (**Figure 3B**). Increased gut pathology that we observed in Abx treated mice is not necessarily contributed by increased fungal burden or persistence of fungal infection in the gut. This was evidenced by PAS staining that did not show much fungal presence in the intestine (**Figure 3A**). We believe that while the initial oral/fungal infection might have triggered the inflammation pathology, loss of Foxp3+ cells and concomitant increase in IFN-γ producers (Th1) and Th1^∗^ cells mainly contributed to gut pathology (**Figures 3C,D**). While SCFA reduced Th1^∗^ cells, they only partially suppressed gut inflammation. This was also associated with a small increase in IL-17A^high^ Th17 cells and T_reg_ cells. However, SCFA significantly decreased overall IFN-γ production, especially in gut Th1^∗^ cells (**Figure 3B**). Thus it appears that generation and control of Th1 and Th1^∗^ cells in the gut likely depends on gut cytokine milieu, distinct from that of oral mucosa. While these results portend that gut and oral mucosa have different mechanisms, our results did not completely rule out the possibility that gut microbiota dybiosis caused some of the changes observed in oral mucosa. Also, it appears that SCFA mediated modulation of IFN-γ might depend on local cytokine milieu and be independent of presence of T_regs_. Future studies, also examining the IFN-γ production by ELISA will clarify these findings. During a short-term acute infection and re-infection in FDTR mice, loss of T_regs_ did cause oral immune pathology, but did not lead to overt colon inflammation (**Figure 5**), showing that long-term Abx treatment, combined with depletion of T_regs_ and repeated long term CA infections are required for gut inflammation (**Figure 3**). Because long-term treatment of Abx alone in un-infected mice did not by itself result in spontaneous inflammation in gut (data not shown), it is clear that a pathogenic infection such as CA infection must be a triggering factor for colon inflammation in the context of microbial dysbiosis and SCFA deprivation. Results from our *in vitro* experiments (**Figure 4**), where SCFA demonstrated diametrically opposite effects on naïve T cells, T cells in whole organ cultures, and mucosal tissue cells, further validate that SCFA has one or more mechanisms of regulating T cells depending on the microenvironment with different cell types and cytokines in the milieu. These inferences are fully consistent with previous studies in colon (Arpaia et al., 2013). With regards to T_regs_, it was clear that SCFA consistently induced an increase in Foxp3+ T cells in all settings, both *in vitro* and *in vivo*, and thus might be the central mechanism by which SCFA mediate their immunomodulatory effects. Our *in vivo* experiments using FDTR mice confirm this tenet (**Figure 5**). In these mice, reduced frequency of Foxp3+ T_regs_ correlated well with the decreased frequency of IL-17A producers and reduced fungal clearance. Although SCFA did not protect T_reg_ depleted mice from weight loss, we found considerably less infiltrating cells in tongue. Reduced inflammation could have been due to small increase in T_regs_ that we observed in these mice (**Figure 6A**). We believe that this effect might also have been mediated by SCFA acting on other cells such as macrophages and dendritic cells (Singh et al., 2014). Although we did not investigate the mechanism by which SCFA mediated T_reg_ induction directly, it was likely dependent on HDAC activity of SCFA that is known to increase Foxp3 protein acetylation and stability (Vinolo et al., 2011; Arpaia et al., 2013). The precise details remain to be seen. Taken together, these results show that during OPC, one or more interdependent T_reg_ populations have multiple layers of protective effects, one for IL-17A induction in CD4 T cells, and another for immunomodulation to prevent excessive inflammation. By enhancing the T_reg_ numbers and possibly functions, SCFA derived from resident bacteria play unequivocal role in promoting protective Th17 cells. Thus our results have identified previously unknown functions of SCFA in Th17 mediated anti-microbial resistance in oral mucosa. Excessive or reduced SCFA may be a sign of commensal dysbiosis and pre-disposition to pathogenic infections and inflammation in humans.

## Author Contributions

PP designed the study, performed experiments, analyzed the data, supervised the project, and wrote and edited the manuscript. NB and CQ performed *in vitro* and *in vivo* experiments, infections, and analyzed ELISA data. SB and CP performed microscopy experiments. ES performed and analyzed ELISA data.

## Conflict of Interest Statement

The authors declare that the research was conducted in the absence of any commercial or financial relationships that could be construed as a potential conflict of interest.

## Abbreviations

Abx: antibiotics
CA: *C. albicans*
FDTR mice: mice expressing diphtheria toxin (DT) receptor in Foxp3+ cells
OPC: oropharyngeal candidiasis
SCFA: short chain fatty acids
T_regs_: Foxp3+ regulatory cells.
