# Supplementary material for: Role of Short Chain Fatty Acids in Controlling Tregs and Immunopathology During Mucosal Infection
Source: Front Microbiol. 2018 Aug 24;9:1995. doi: 10.3389/fmicb.2018.01995 (PMC6117408; doi:10.3389/fmicb.2018.01995)
Supplement: Supplementary file 1 [file Data_Sheet_1.PDF]

Supplementary figures

Figure.S1

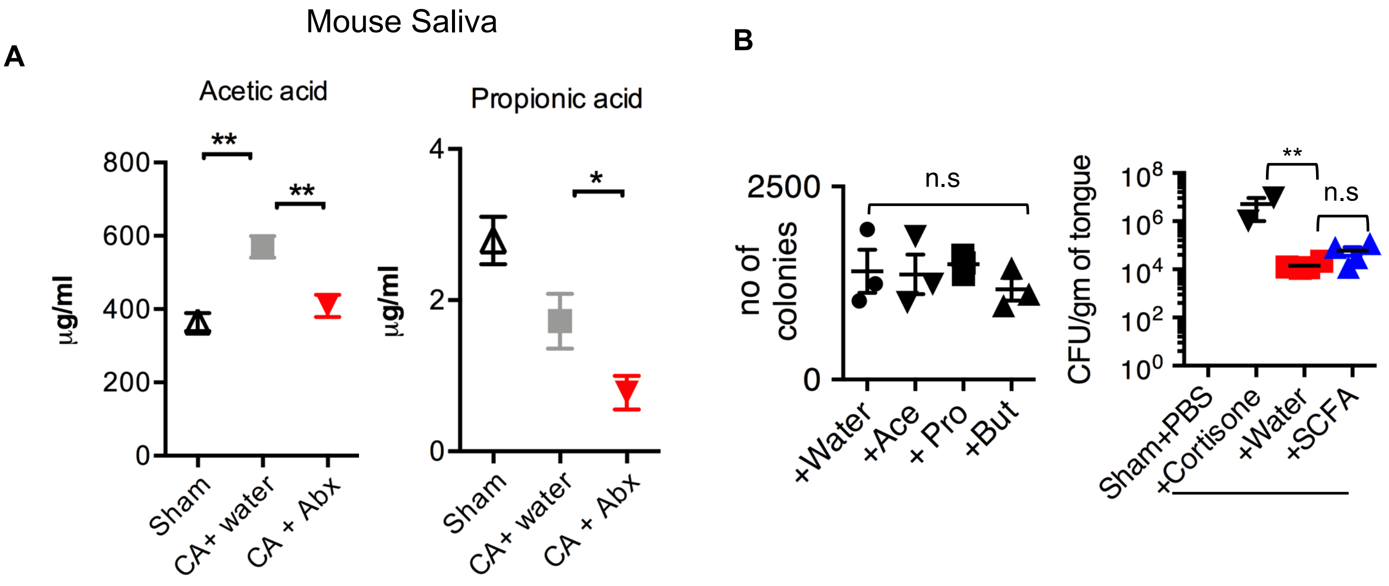

Fig.S1. A) Abx lowers salivary SCFA levels in mice. Acetate and Propionate were estimated by Gas chromatography-Mass spectroscopy (Commercial service), using pooled saliva that was collected on days 0, 7, 14, and 35 after secondary infection. Other SCFAs such as butyrate, iso-butyrate and valerate were below detectable levels, and thus the data are not shown. **B) SCFAs have no impact on CA growth *in vitro*, and during infection of normal mice *in vivo*.** *C.albicans* blastospore cultures grown overnight were mixed at serial dilutions (undiluted, 1:10 and 1:100) with pH equalized water, 10 mM (Acetate), 1mM (Propionate), 0.5 mM (Butyrate) and plated in Sabaroud dextrose agar. Colonies were counted at 36 hours after incubation. incubation (left). Fungal colony forming units (CFU) in the tongue of the infected mice (normal mice with no antibiotics) on day 3 after *C.albicans* infection. Cocktail of propionate (50mM) and butyrate (50mM) were given in drinking water. (n.s = non significant; ** P < 0.005) (right).

Figure.S2

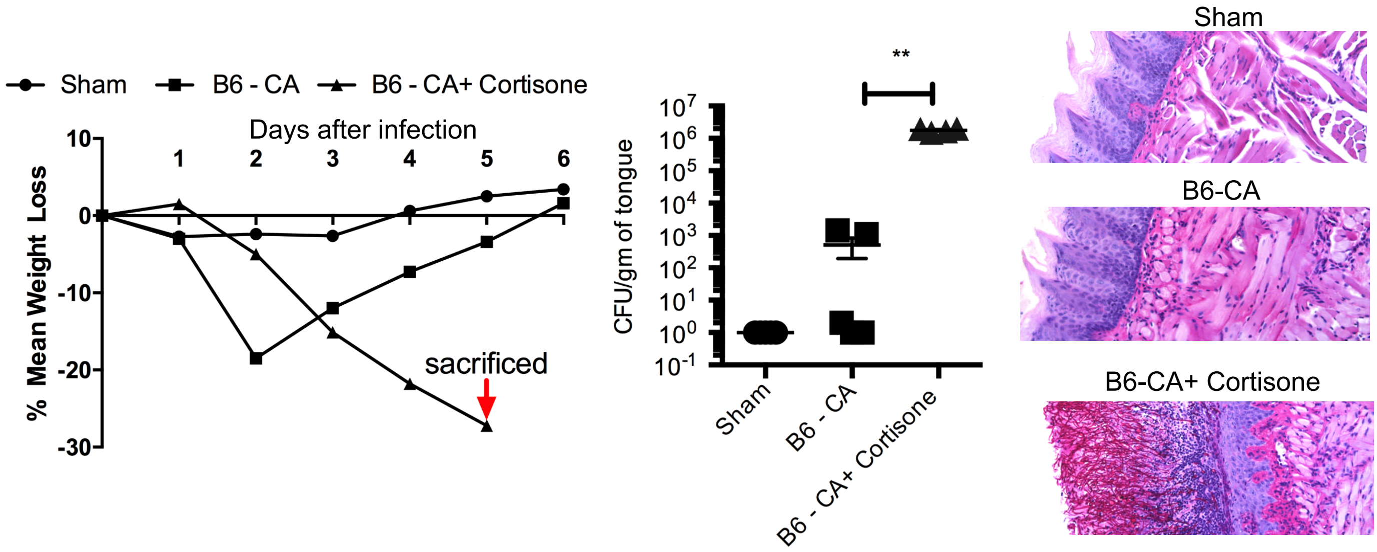

Fig. S2. Cortisone causes severe OPC disease in mice. Mice were infected as in Fig.1 without or with Cortisone. For cortisone (immunosuppressed) group, cortisone acetate was administered at a concentration of 225 mg kg−1 in a total volume of 0.2 ml of PBS. No reinfection was done in this experiment. The percent weight change in mice with respect to d0 of primary infection(left). On day 5 or 7 after infection, tongues were harvested and fungal burden was determined in tongue lysates (middle), or by PAS staining (right).

Figure.S3

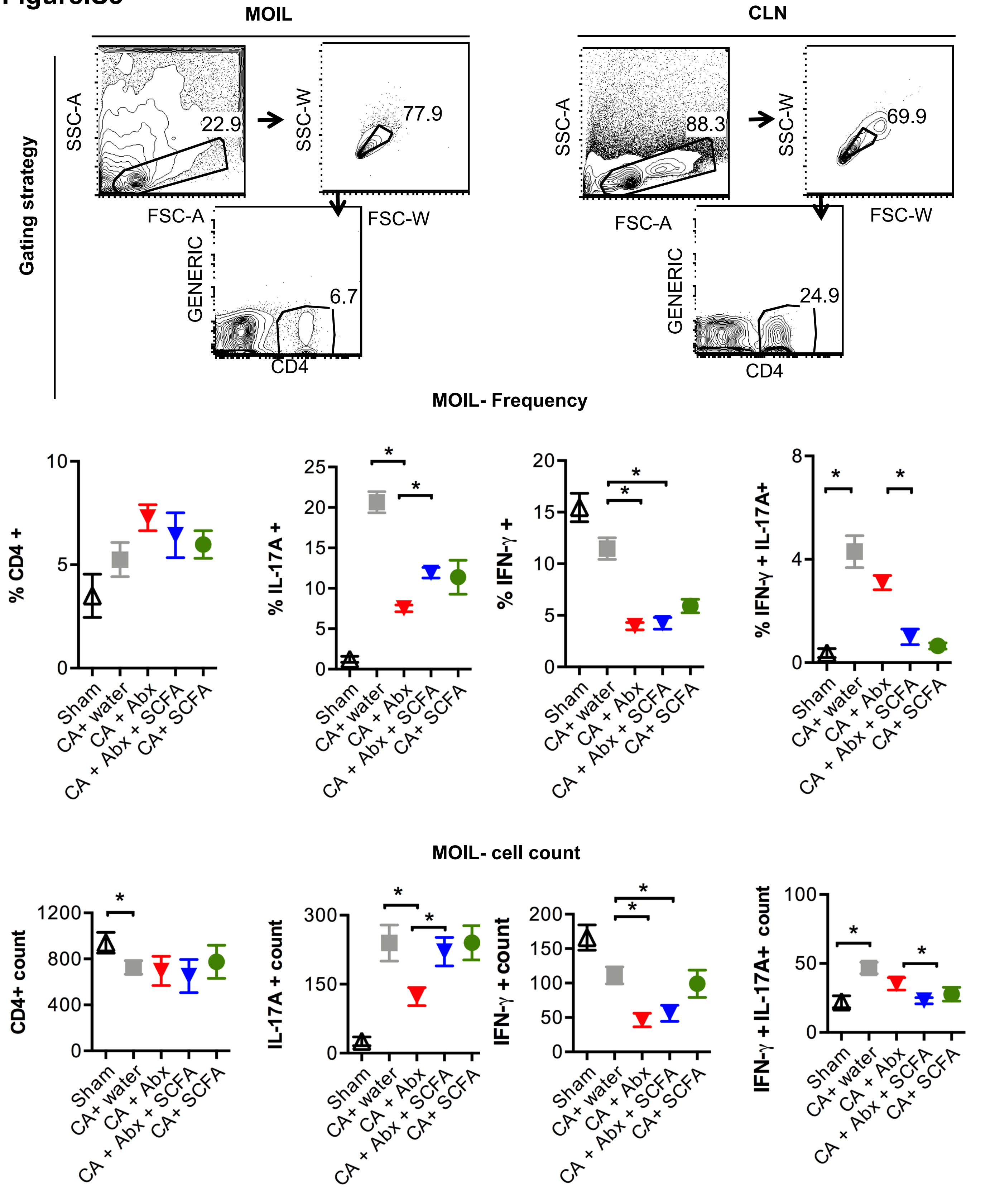

Fig.S3. Abx treatment leads to Th17 and Th1 cell reduction, and SCFA administration increases IL17A+, cells but not Th1 cells in oral mucosa during OPC. Mice were treated with Abx and SCFA, and infected as in Fig.2 (n=4/group). On day 1 after re-infection, cells isolated from tongue (MOIL; left) and CLN (right) were re-stimulated for flow cytomtery. Gating strategy is shown (top panel). Statistical analyses of frequency and cell counts were done using Mann-Whitney test (*P < 0.05) (below). Data represent one out of three experiments.

Figure.S4

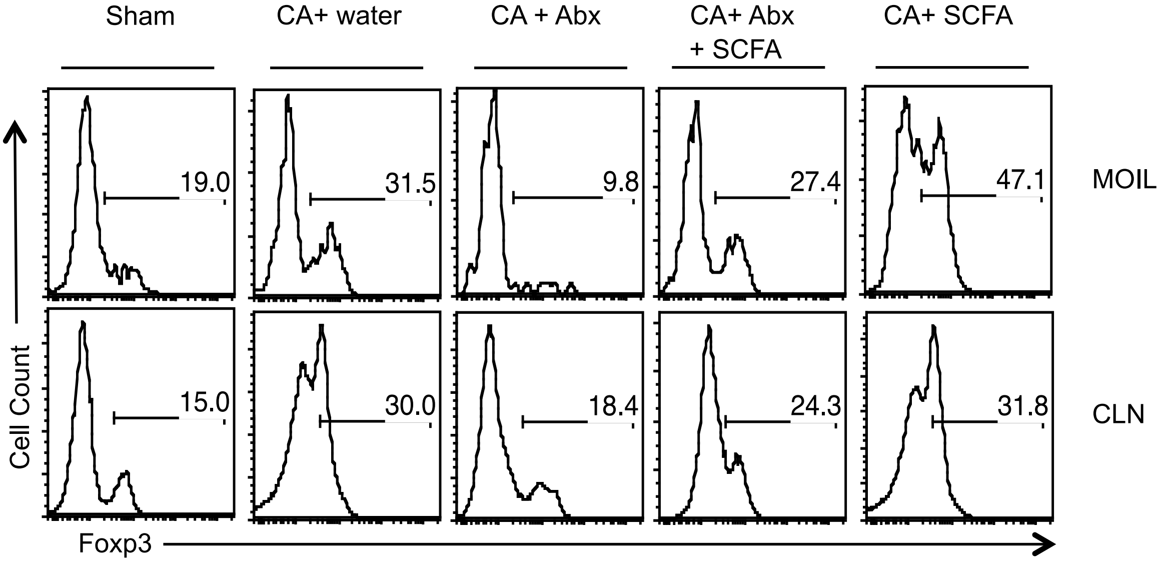

Fig.S4. Abx treatment diminishes Foxp3+ cells, and SCFA administration restores these cells in oral mucosa during OPC. Mice were treated with Abx and SCFA, and infected as in Fig.2 (n=4/group). On day 1 after re-infection, cells isolated from tongue (MOIL; left) and CLN (right) were re-stimulated for flow cytomtery.

Figure.S5

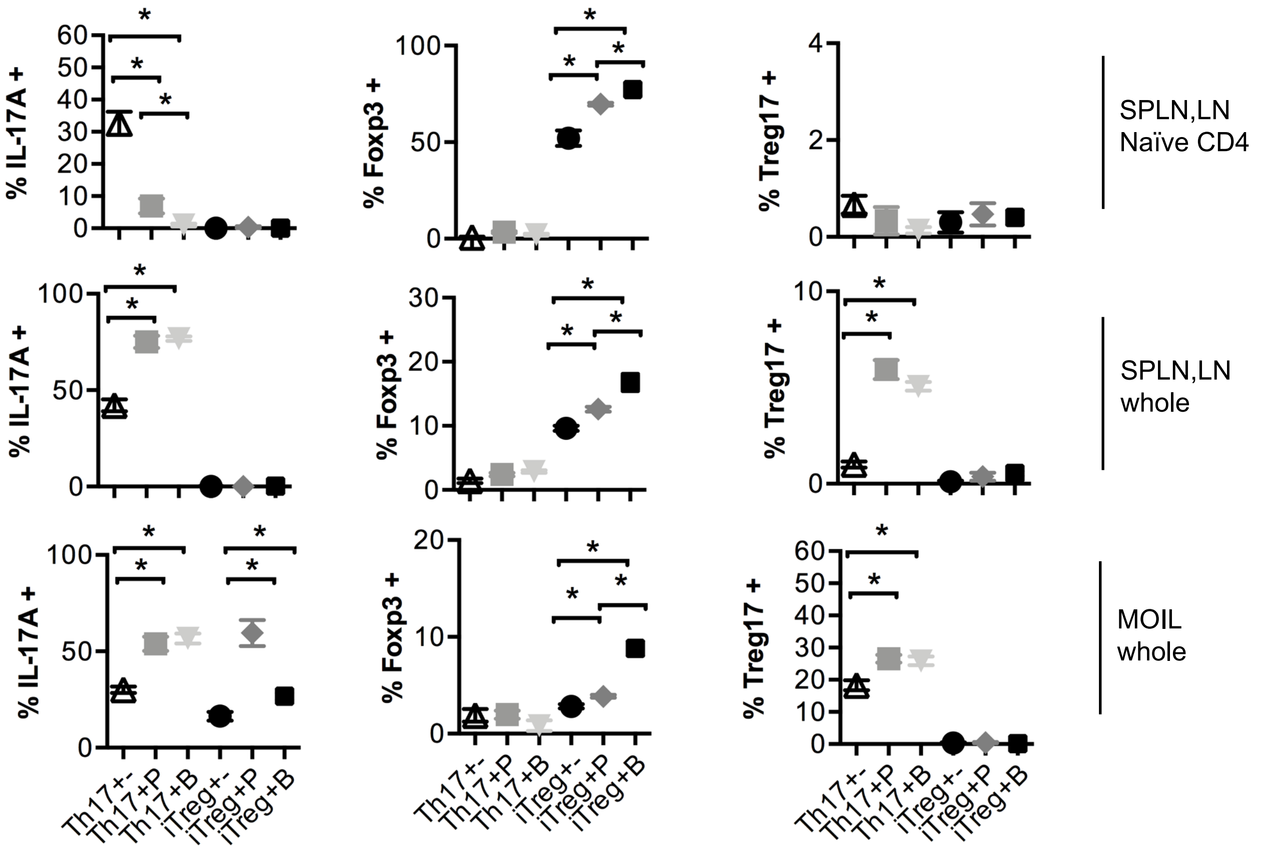

Fig.S5. SCFAs promote IL-17A, Foxp3 expression, and cells co-expressing IL-17A and Foxp3 (Treg17), depending on cytokine milieu *in vitro.* Naïve cells from normal WT mice were isolated from pooled SPLN and all lymph nodes(LN), or MOILs as in Fig.4, and stimulated under Th17 or iTreg conditions with or without propionate (P) and butyrate (B). IL-17A and Foxp3 expression (in CD4+ cells) were assessed by flow cytometry on day 4 after stimulation.

Figure.S6

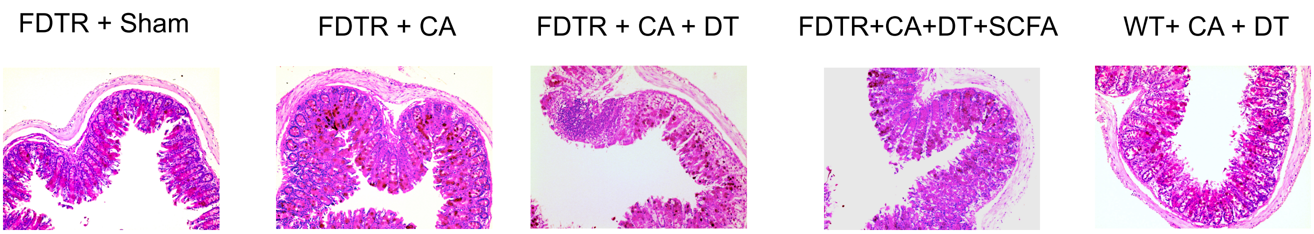

Fig.S6. Loss of T_{regs} or SCFA do not result in colon inflammation in FDTR mice during short-term acute CA infection. FDTR and WT mice were pre-injected with DT, or pretreated with SCFA as in Fig.5 (n= 3-4/ group). Colon PAS staining was performed to assess fungal burden, mucin secretion and inflammation (200X magnification). 3 independent experiments show similar results.

Figure.S7

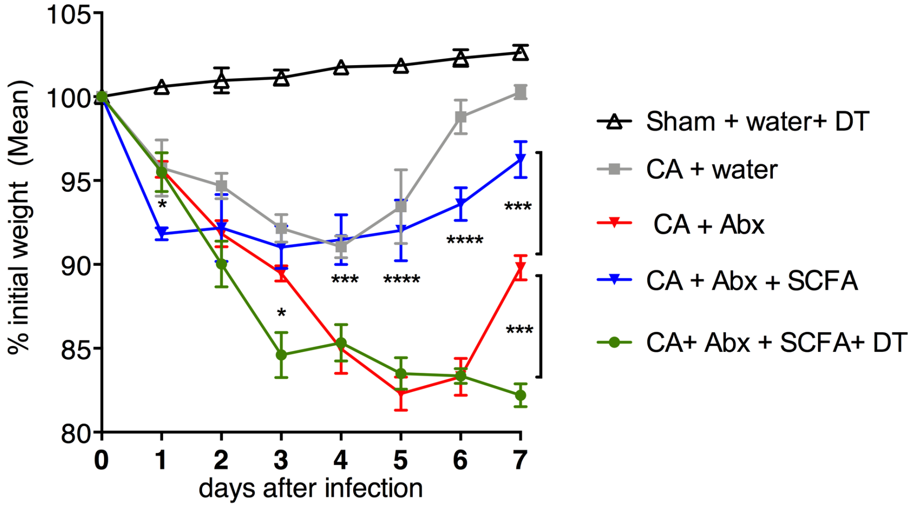

Fig.S7. SCFA administration provides no protection to Abx treated FDTR mice in the absence of T_{regs} during short-term acute CA infection. FDTR mice that were pre-treated with Abx and SCFA as in Fig.1, were injected with DT (n= 3-4/ group) and infected with CA. The percent weight change in mice with respect to d0 of primary infection. Statistical analyses were done using 2-way ANOVA test (*$P < 0.05$). Data represent duplicate experiments.
